# The Effect of Toothpaste Suspensions on the Formation of Oral Biofilms In Situ

**DOI:** 10.1016/j.identj.2025.104018

**Published:** 2025-11-23

**Authors:** Madline Priska Gund, Lea Lehnertz, Matthias Hannig, Johanna Dudek, Norbert Pütz, Stefan Rupf

**Affiliations:** a Clinic of Operative Dentistry, Periodontology and Preventive Dentistry, Saarland University Medical Centre, Homburg/Saar, Germany; b Synoptic Dentistry, Saarland University, Homburg/Saar, Germany

**Keywords:** Tooth paste, Biofilm formation, Vital fluorescence, Scanning electron microscopy

## Abstract

**Objectives:**

Toothpastes are essential for oral hygiene and plaque control, yet limited data exist on their ability to inhibit biofilm formation. This study evaluated the biofilm-inhibiting effects of four toothpastes using an in situ biofilm model.

**Design:**

Paste I contained fluoride, chlorhexidine, and aluminum lactate. Paste II added strontium acetate and potassium chloride to Paste I. Paste III included hydroxyapatite, and Paste IV contained papain and bromelain. Controls included water, a base paste, and 0.2% chlorhexidine. Six volunteers wore acrylic splints with bovine enamel slabs and rinsed with toothpaste suspensions at intervals over 48 hours. Biofilm coverage and bacterial viability were assessed via fluorescence microscopy using live/dead staining. Results: Results showed reduced biofilm coverage with Paste I (31%*), Paste II (29%*), Paste IV (30%*), and Paste III (40%) compared to water (72%) and base paste (47%). Chlorhexidine showed the lowest coverage (10%). Viability of bacteria (green fluorescence) was lowest with Paste III (9%*), followed by Paste I (31%*), Paste II (28%*), and Paste IV and chlorhexidine (each 39%). Water and base paste showed higher viability (54% and 51%, respectively). Statistically significant differences were found compared to water (Mann-Whitney U-test, *P < .05).

**Conclusions:**

Toothpastes with active ingredients significantly reduced biofilm formation and/or viability. Paste III, containing hydroxyapatite, was particularly effective in reducing bacterial viability. Further research is needed to explore alternatives to chlorhexidine as a preservative.

## Introduction

According to the World Health Organization (WHO) Oral Health Status Report from 2022, 3.5 billion people worldwide are affected by oral diseases. The 2019 Global Burden of Disease Report identifies untreated tooth decay as one of the most prevalent health issues worldwide.^1^ While these conditions are rarely life-threatening, they can cause significant stress and discomfort for those impacted. Over the past century, significant advancements have been made in understanding oral microbiology, although many aspects remain under investigation. A key focus in this field is the dental biofilm, a complex bacterial community composed of various types of bacteria embedded within an extracellular matrix.^2^ Biofilm formation initiates with pellicle formation, created by the electrostatic attachment of adsorbed proteins, enzymes, and glycoproteins from saliva. This process involves a non-specific reversible interaction between the pellicle layer and microorganisms, followed by a phase of irreversible interaction mediated by bacterial adhesins. Once individual bacteria adhere, co-adhesion occurs, allowing additional bacteria to adhere and form colonies. As the biofilm matures, it develops horizontal and vertical layers, resulting in extensive colonization.^3^^,^^4^

Plaque control primarily aims to prevent the colonization of cariogenic bacteria, which produce acids that lower pH within the biofilm and demineralize enamel. Maintaining physiological conditions refers to maintaining a neutral pH and a balanced microbial ecosystem in the oral cavity.^5^ However, it should be complemented by chemical agents.^6^ A variety of antimicrobial agents are available for biofilm control, often used in toothpastes or mouthwashes.^7^ The development of modern toothpastes can be traced back to the 19th century, with continuous improvements since then. The ingredients are divided into active and inactive categories. Active ingredients are therapeutic agents that vary depending on the area of application. Inactive ingredients include substances responsible for shelf life, taste, rheology, abrasion, foaming, and colour, among others.^8^

One of the most important agents for plaque control is the polybiguanide chlorhexidine (CHX). Its positive charge allows it to bind to negatively charged surfaces, altering bacterial membrane integrity. Depending on concentration, it can exhibit either bacteriostatic or bactericidal effects. However, long-term use may result in discoloration and sensory disturbances, as well as a burning sensation in the mouth and tongue, and in some cases, pain and swelling in the oral cavity.^9^^,^^10^ Another ingredient is aluminum lactate, a salt of lactic acid known for its protein-coagulating, astringent, and haemostatic properties.^11^

Fluorides, widely recognized as essential components in oral hygiene products, have played a significant role in the global reduction of caries. Present in both the environment and all living organisms, these salts of hydrofluoric acid promote the diffusion of fluoride intro various oral components, including the saliva, pellicle, microbial biofilm, dental hard tissue and oral mucosa.^12^^,^^13^ Fluorides are considered the most important substances in caries prevention, serving as a benchmark against which comparable remineralization systems are measured.^14^ Extensive data demonstrates the vital role of fluoride in caries prevention.^15^

Hydroxyapatite (HAP) is an inorganic material, with the molecular formula Ca5(OH)(PO4)3 which possesses a unique lattice structure.^16^ As a nanoparticle approximately 20 nm in size, it can interact with both the enamel surface and microorganisms.^17^^,^^18^ Research suggests that a concentration of 10% HAP may optimize its remineralization potential.^19^ While previous studies have demonstrated that hydroxyapatite can promote the remineralization of early carious lesions and prevent demineralization,^20^ its sole protective effects against caries remain a topic of debate. Several studies and meta-analyses have investigated the comparative efficacy of fluorides and hydroxyapatite, yielding mixed results. For instance, Wierichs found no definitive evidence supporting the effectiveness of nano-HAP.^21^ In contrast, reviews by Anil and Limeback highlight the positive therapeutic and preventive effects of hydroxyapatite, noting that most studies reported beneficial outcomes related to remineralization, reduced demineralization, and caries prevention.^22^ Specifically, Limeback demonstrated a notably lower incidence of caries associated with hydroxyapatite, particularly in paediatric populations.^23^

For the treatment of hypersensitivity, toothpastes containing potassium chloride and strontium acetate are frequently used. Potassium chloride, a potassium salt of hydrochloric acid, has been shown to significantly reduce hypersensitivity by suppressing the response of A-beta and A-delta fibres. A-beta fibres transmit non-painful stimuli, such as light touch, while A-delta fibres carry sharp, localized pain signals in response to harmful stimuli, including pressure and extreme temperatures.^24^ Strontium acetate, a strontium salt of acetic acid, works by occluding tubules. It can significantly reduce dental hypersensitivity and thus provide pain relief.^25^

Enzymes such as papain and bromelain, derived from fruits, have been utilized in medical applications for decades and are now being incorporated into whitening toothpastes.^26^ Bromelain is a cysteine protease extracted from the pineapple catalysing the cleavage of proteins into amino acids.^27^ It has fibrinolytic, antithrombotic, anti-inflammatory and anti-oedematous properties. Papain, extracted from the papaya fruit, similarly breaks down organic molecules into amino acids.^28^

Hyaluronic acid (HA) is a high molecular weight glycosaminoglycan that occurs naturally as a component of the extracellular membrane of connective tissue, synovial fluid, and other tissues.^29^ It has been used in medicine, pharmacology and cosmetics for many years. Known for its anti-inflammatory properties, HA also displays anti-edematous and antioxidant capabilities. Hyaluronic acid can bind prostaglandins, metalloproteinases and other bioactive molecules, which explains its anti-inflammatory effect.^30^ A recent in vitro study suggests that HA can inhibit biofilm formation and the growth of Porphyromonas gingivalis, a major pathogen in periodontal disease, demonstrating effects comparable to CHX.^31^

The present study aimed to investigate the effects of different toothpastes on the biofilm in a 48 h in situ biofilm model. We used toothpaste suspensions to ensure a controlled concentration of active ingredients, allowing the effect to be studied without mechanical cleaning. This approach addresses the lack of studies on the antimicrobial activity of toothpastes against the formation of oral biofilms.

## Materials and methods

### Subjects and specimens

Six volunteers (n=6) working in the field of dentistry, either as dental students or as employees of the Department of Operative Dentistry, Periodontology and Preventive Dentistry, Saarland University Hospital, Homburg, Germany, were included in the study. All participants had acceptable oral hygiene and health, without caries cavities and healthy periodontal conditions (bleeding on probing < 10 %). None had received antibiotic treatment within the previous 6 months.

A total of 168 enamel slabs were prepared from bovine incisors of 2-year-old cattle. The crowns of the teeth were cut into rectangular specimens (5 × 5 × 1 mm) using a circular saw and wet grinding machine (Buehler, Düsseldorf, Germany). Specimens were polished up to 2.500 grit using silicon carbide grinding paper (Buehler, Duesseldorf, Germany) and disinfected by ultrasonication for 3 min in 3 % sodium hypochlorite and 15 min in 70 % isopropanol. Finally, specimens were stored in sterile water for 6 h. Two specimens were fixed to individual left and right upper jaw splints made of methacrylate (Scheu Dental, Iserlohn, Germany) using silicone impression material (Coltène/Whaledent, Langenau, Germany).^32^ One of the enamel slabs from left and right splints were used for fluorescence microscopy, the other for scanning electron microscopy.

The Ethics Committee of the Saarland Medical Association approved the trial protocol and declared that there were neither professional nor ethical concerns about the study (ethics vote no. 101/22, extended no. 198/22). The study was conducted according to the guidelines of the Declaration of Helsinki.

### Test substances

The subjects rinsed with suspensions of four active toothpastes and one basic toothpaste without active ingredients. Water and basic toothpaste served as negative controls, while CHX (0.2%) was used as a positive control. Water (Ampuwa®, Fresenius Kabi, Bad Homburg, Germany) and CHX (Chlorhexidine 0.2 %-digluconate solution, Saarland University Pharmacy, Homburg, Germany) were provided as ready-to-use substances. The toothpastes were supplied by Dr Theiss Naturwaren GmbH. A suspension of 0.5 g paste and of 2 ml sterile water was prepared from the toothpastes for rinsing. The toothpastes and their ingredients are listed in the Table.

**Table:** Test substances and toothpastes used in the present study

Test substance	Components	Experimental role
base paste	aqua, polyethylene glycol 32 (PEG), sorbitol, hydroxyethyl cellulose, silica, titanium dioxide, sodium lauryl sulphate (SLS)	Negative control
standard paste	Base paste + sodium fluoride 1450 ppm, aluminum lactate (0.8 %), chlorhexidine (CHX (0.05 %))	Test substance: paste I
sensitive paste	Base paste + sodium fluoride 1450 ppm, aluminum lactate (0.8 %), CHX (0.05 %), strontium acetate (2 %), potassium chloride (3 %),	Test substance: paste II
hydroxyapatite paste	Base paste + sodium fluoride, sodium fluoride 1450 ppm, aluminum lactate (0.8 %), CHX (0.05 %), hydroxyapatite (5 %),	Test substance: paste III
enzyme paste	Base paste + sodium fluoride 1450 ppm, aluminum lactate (0.8 %), CHX (0.05 %), EDTA, papain (0.012 %), bromelain (0.05 %), calcium glycerophosphate, hyaluronic acid (0.2 %), magnesium hydrogen citrate	Test substance: paste IV
chlorhexidine (CHX)	chlorhexidine-digluconate 0.2 %	Positive control
sterile water	H2O	Negative control

### Study design

Preliminary tests were carried out with each volunteer, in which the individual biofilm formation on enamel test specimens was examined and the adaptation to the test procedures was checked. These tests were carried out in order to have participants with comparable biofilm formation available and to avoid problems with correct adherence to the test procedure. Each participant wore the splints with enamel slabs a total of 7 times for 48 h each, starting with water, followed by basic toothpaste, toothpastes I to IV and, finally CHX. A wash-out phase of 14 days was kept between the test cycles, during which the participants brushed with the following toothpaste. Before the experiment with CHX, the participants continued to use paste IV. Mouth rinses were not used during the entire period. The splints were worn in the oral cavity for 48 hours. Participants rinsed their oral cavity with the toothpaste suspensions, water or CHX thoroughly for 60 s at 30 min, 12 h, 24 h, and 36 h after placing the splints. When eating or drinking anything else than water, subjects kept the splint in a moist environment and reinserted it after 30 min latest. Teeth brushing after splint removal was limited to the use of water only. At the end of the 48-hour wearing period, the splints were removed from the oral cavity and the enamel slabs were further processed.

### Vital fluorescence

One specimen from the splint on each side was used for fluorescence microscopic analysis. The LIVE/DEAD® BacLightTM Bacterial Viability Kit (Invitrogen, Molecular Probes, Eugene, Oregon, USA) was used to stain the biofilms on the enamel slabs. After staining with 10 µl of the dye solution composed of 1 µl SYTO9 and 1 µl propidium iodide in 1000 µl of a 0.9% sodium chloride, samples were incubated in a dark chamber for at least 10 minutes. For each specimen six images were captured at a magnification of 1000x using a fluorescence microscope (Axio Skop, Carl Zeiss AG, Oberkochen, Germany) and program Axio Imager M2. Finally, the ImageJ software (Image J 1.52, NIH, Bethesda, Maryland, USA) was used to analyse coverage and vitality of the biofilms.

Coverage refers to the percentage of the area occupied by biofilms in one image. In the analysis using ImageJ, coverage is commonly calculated by comparing the number of pixels of the biofilm occupied area to the total area of the image. Vitality, on the other hand, pertains to the proportion of green fluorescence in comparison to red fluorescence.

### Scanning electron microscopy

The two remaining enamel slabs were used for scanning electron microscopy (SEM) to assess the morphology of the biofilm and to detect toothpaste components present in the biofilms. Fixation was performed in 2% glutaraldehyde in 0.1 M cacodylate buffer for at least one hour at 4°C with evaporation protection. The samples were washed three times with 0.1 M cacodylate buffer at room temperature for 10 minutes, followed by dehydration using an ascending series of alcohol at room temperature. Afterwards, the samples were left overnight to dry, sputtered with carbon and were then analysed in a scanning electron microscope (XL 30 ESEM FEG, FEI Company, Eindhoven, Netherlands) at 25x to 20.000x magnification followed by energy dispersive X-ray spectroscopy (EDX).

### Statistics

Statistical analysis was performed using the GraphPad Prism10 program (GraphPad Software, San Diego, California, USA). The statistical analyses were carried out against two substances, water as a negative control and the basic toothpaste as a suspension without active ingredients using the Mann-Whitney U-test. *P*-values < .05 were considered statistically significant.

## Results

All volunteers completed the experiments without complications.

### Fluorescence microscopy

**Fig. 1:**
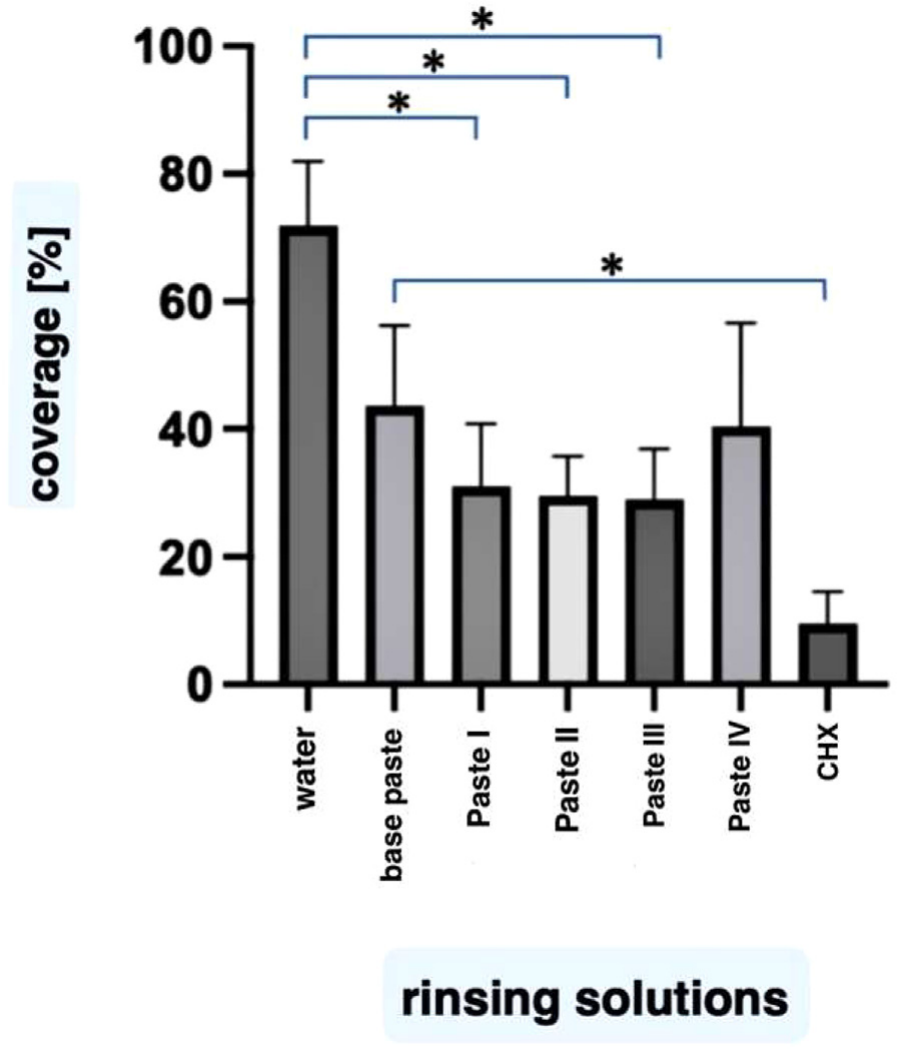
The coverage of enamel surfaces with biofilms after the in situ experiments shown in %. The height of the bars corresponds to the mean, the whiskers correspond to the ± standard deviations. Mean values are expressed as % of the test surface covered by biofilms. The differences between water and paste I, paste II, paste IV and CHX were statistically significant (*P* < .05). CHX also showed a significant reduction (*P* < .05) compared to the base paste. Significant differences (*P* < .05) are marked with * in the graph.

The highest percentage of biofilm covering the specimens was measured for water at 79 ± 11 %. The basic toothpaste resulted in 47 ± 28 % coverage. The positive control CHX reduced the coverage to 10 ± 12 %. The toothpaste suspensions containing active ingredients reduced to 31 ± 24 % for toothpaste I, to 29 ± 19 % for toothpaste II, to 40 ± 40 % for toothpaste III and to 30 ± 15 % for toothpaste IV. Compared to water, a statistically significant reduction of biofilm formation was observed with paste I (*P* = .01), paste II (*P* = .005), paste IV (*P* = .005) and CHX (*P* = .005). The results for the basic toothpaste (*P* = .6) and paste III (*P* = .15) were not statistically significant in comparison to water. When comparing the results of the basic toothpaste with the other pastes, a statistically significant reduction was only observed for CHX (*P* = .01) (Figure 1).

### Vital fluorescence

**Fig. 2:**
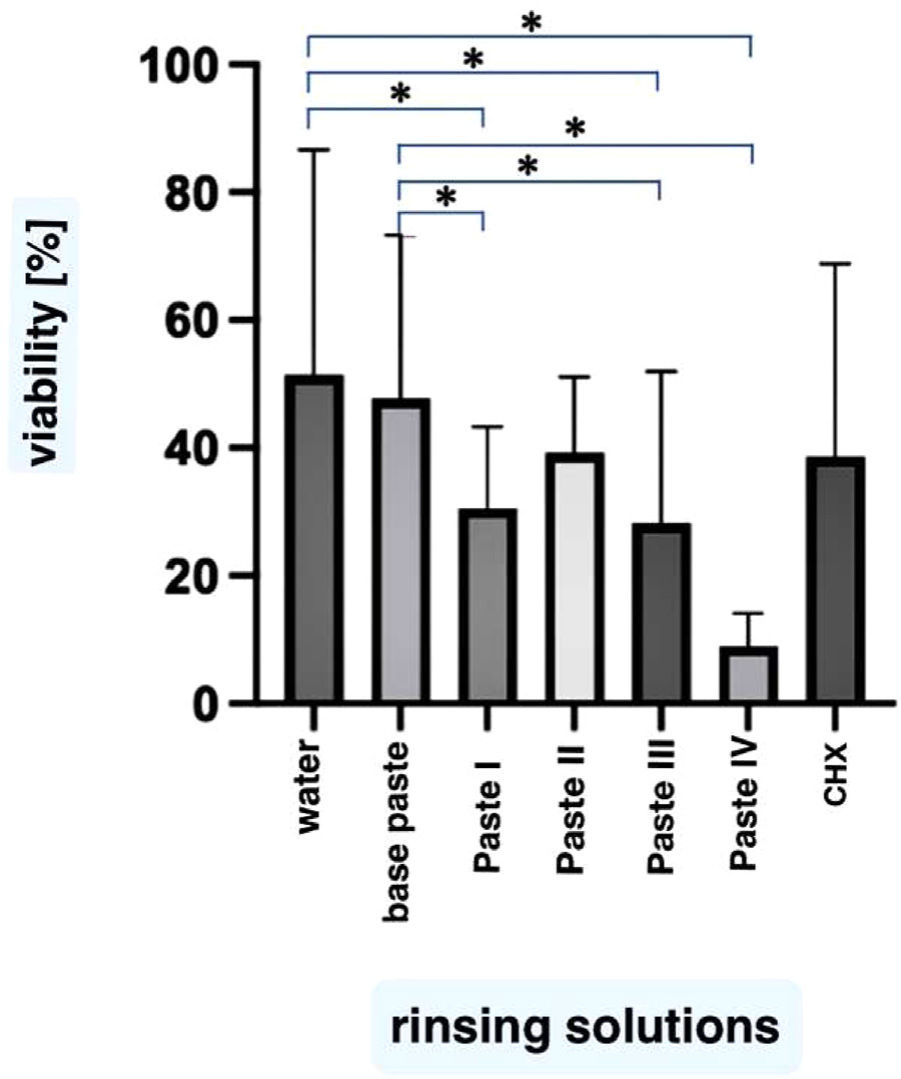
The green fluorescence of biofilms as percentage of the overall fluorescence (green plus red) representing vital bacteria in the biofilms. Vitality of biofilms according to the individual rinsing solutions is shown in %. The height of the bars corresponds to the mean, the whiskers correspond to the ± standard deviations. Mean values are expressed as % of the test surface covered by biofilms. The differences between water and paste I, paste III and paste IV were statistically significant (*P* < .05). Statistically significant differences were also found between the results for basic paste vs. paste I and IV.

**Fig. 3:**
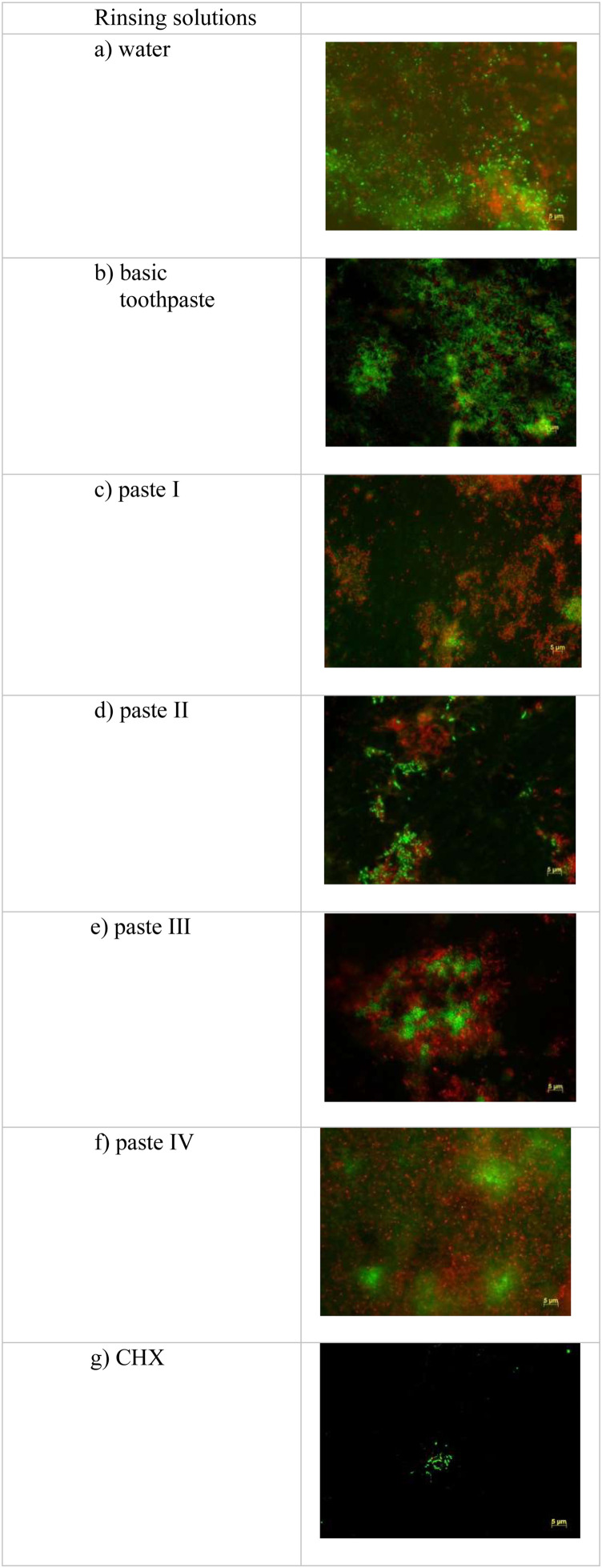
Representative fluorescence microscopy images: the images were taken with the microscope after 48 h of wear after staining the biofilm with the LIVE/DEAD® BacLightTM Bacterial Viability Kit.

The percentage of green fluorescence (vital bacteria) in correlation to red fluorescence (dead bacteria) showed the highest values for water (51 ± 20 %) and the basic toothpaste (47 ± 14 %) without statistically significant differences. The share of green fluorescence was lower in comparison to water for paste I (31 ± 9 %, *P* = .04), for paste III (28 ± 18 %, *P* = .04) and for paste IV (9 ± 3 %, *P* = .005). The green fluorescence for paste II (39 ± 7%, *P* > .05) and CHX (39 ± 16%, *P* > .05) were not statistically significantly different from water. In comparison to the basic toothpaste, the results for paste I (*P* = .04) and paste IV (*P* = .005) differed statistically significantly (Fig. 2, Fig. 3).

### SEM and EDX analyses

**Fig. 4:**
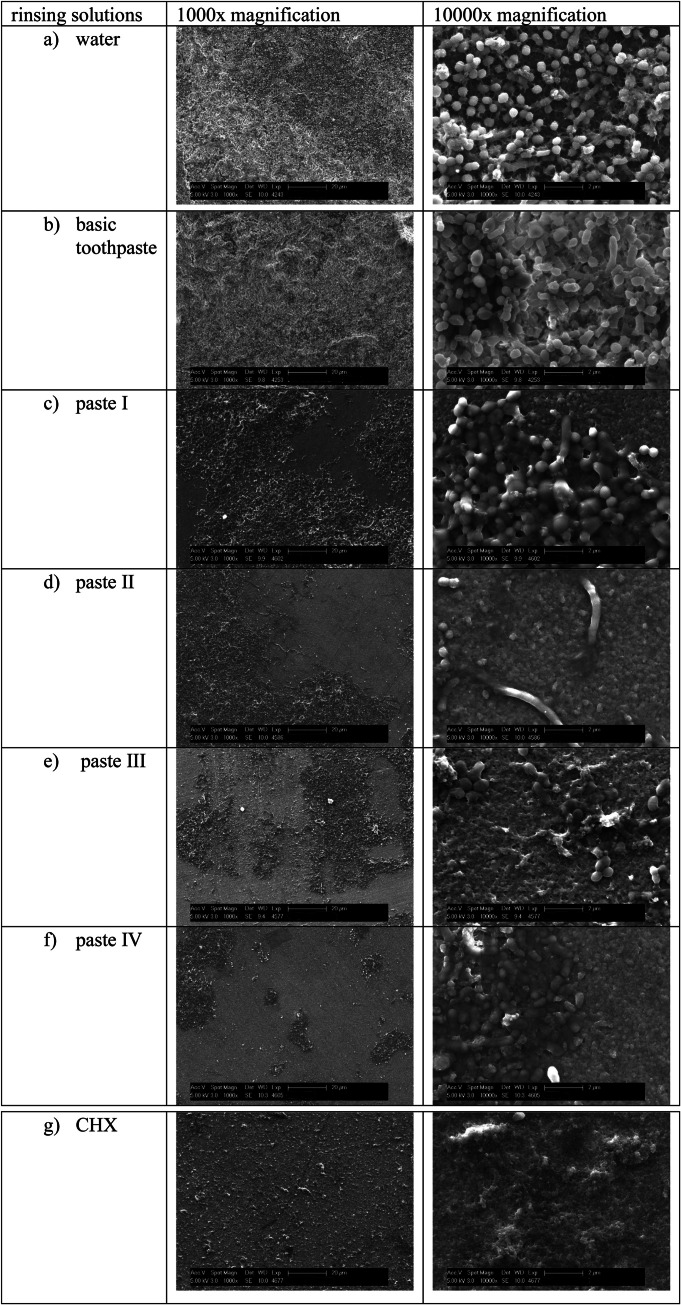
Representative scanning electron microscope images after rinsing at 1000x and 10000x magnification.

Representative images of biofilms confronted with the different substances and toothpastes used in this study are presented in Figure 4. In all biofilms cocci-form bacteria were observed, some organized in chains or colonies. Occasionally, rod-shaped bacteria were visible. Residues of the toothpastes were visible frequently. After both, rinsing with water and the basic paste a multi-layered biofilm and intact bacteria were present. Application of paste I revealed the presence of mostly intact bacterial agglomerates and areas covered only by the pellicle. After using paste II, the biofilm morphology was disrupted, with large areas covered only by the pellicle and the bacteria showing an altered morphology. After paste III, smaller bacteria were visible in comparison to the other pastes. The biofilms confronted with paste IV showed a masked biofilm structure. The bacterial layers were covered with a substance. Only a few rods and cocci penetrated this matrix and were clearly recognizable on the surface. Rinsing with CHX destroyed the biofilm structure, resulting in large areas free of bacteria and remaining bacteria with disrupted morphology.

**Fig. 5:**
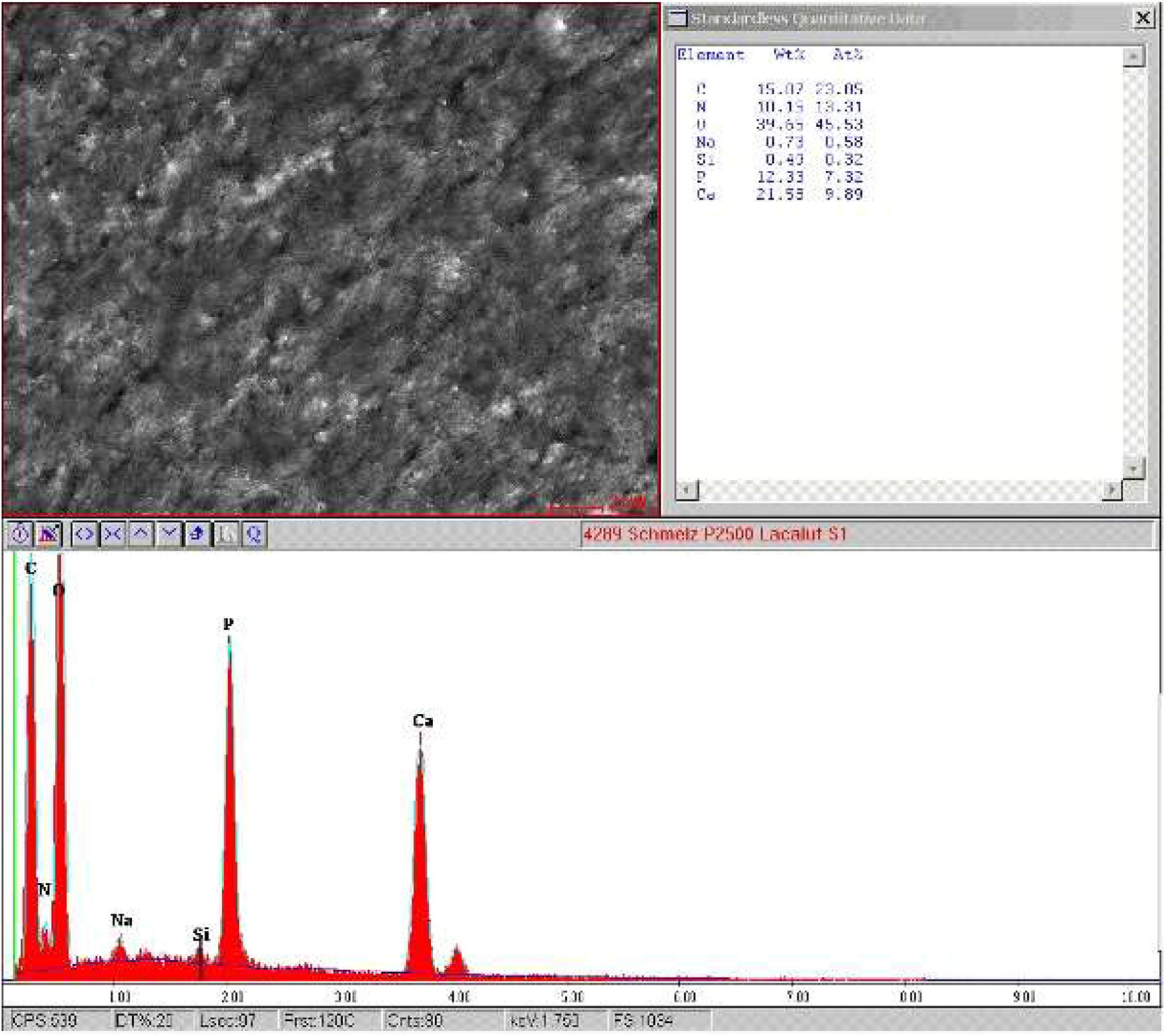
EDX analysis after rinsing with the HAP-P.

**Fig. A1:**
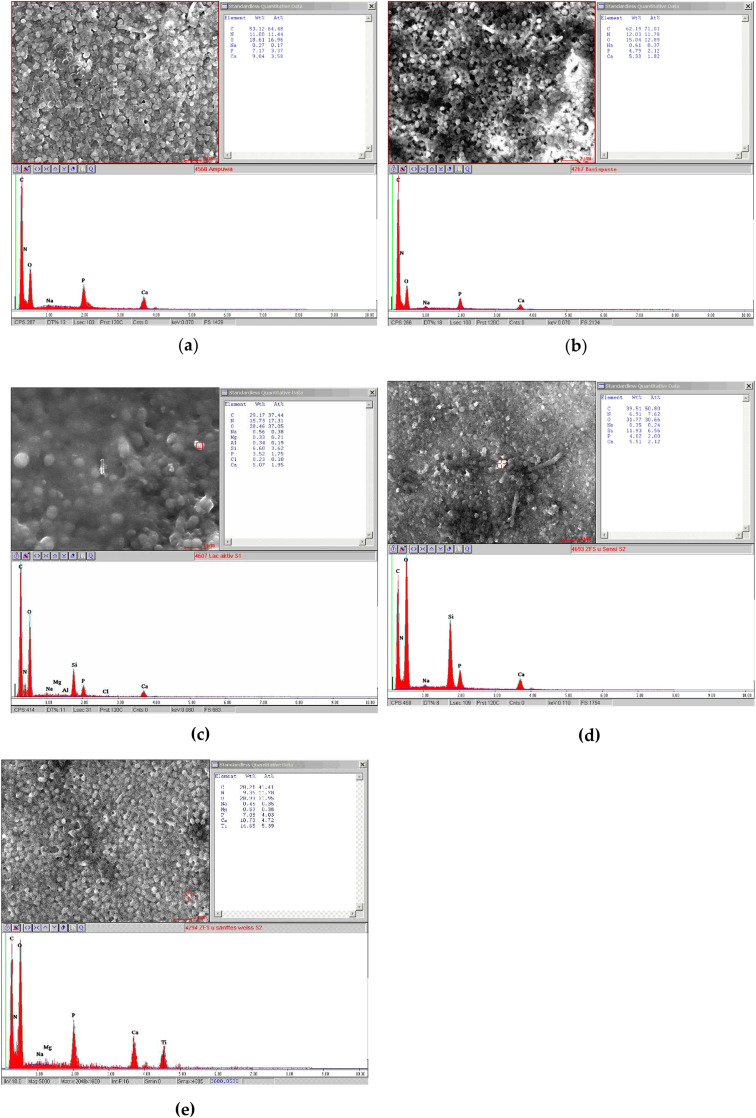
EDX Analysis (a) EDX area analysis after rinsing with water (b) EDX area analysis after rinsing with base paste, no indication of paste residues on the test specimens (c) EDX analysis after rinsing with standard paste, the spot analysis revealed an increased proportion of oxygen (38 % by weight (wt)) and aluminum (0.34% by weight). The elevated oxygen and aluminum content may suggest the potential presence of aluminum lactate in the rinsing solution. The elevated silicon content of 7 wt.-% suggests the presence of residual abrasive substances in the toothpaste (d) EDX analysis following rinsing with the sensitive paste reveals an elevated value of silicon, with a concentration of 12 wt.-% (e) EDX analysis after rinsing with the enzyme paste, an increased proportion of titanium (15% by weight) and oxygen (29% by weight) was detected by the spot analysis, which can be attributed to the presence of toothpaste.

EDX analysis revealed the presence of residues of the toothpastes on the biofilms, which can be attributed to cleaning agents as silicon particles and titanium dioxide. These residues were detected sporadically after using paste I and II (Figure A1). Additionally, aluminum was found, which confirmed the presence of aluminum lactate in the toothpastes (Figure A1). For paste III, residues of hydroxyapatite were found (Figure 5). For paste IV, mainly titanium particles were identified (Figure A1).

## Discussion

The aim of this study was to investigate the effects of toothpastes containing different active ingredients on the new formation of oral biofilms. Previous studies have established the efficacy of single substances like CHX^33^ and hydroxyapatite.^34^ However, there is still an ongoing discussion, if toothpastes per se are able to reduce the biofilm formation after toothbrushing. This investigation performed an in situ approach, using suspensions of toothpastes to study the effects of them on the reduction of dental biofilm formation without the influence of brushing. In order to ensure the reliability of this in situ experiment, a number of measures were implemented to control influencing factors.

A 14-day wash-out or adaptation phase was carried out between the different substances in order to rule out any interference between the toothpastes and substances. The 48-hour duration of biofilm formation ensured sufficient biofilm development on the one hand and, at the same time, repeated influencing of biofilm formation by the test substances. In addition, the duration of the in situ experiment was not too long and the protocol was not too complex, so that the volunteers were able to follow the instructions properly. The six volunteers were dental students or dentists without carious lesions and active periodontal diseases. The professional knowledge and the direct contact with the study leaders thus also ensured a basic awareness of the importance of the adherence to the study protocol. Although this small sample may limit the generalizability of our results, it is comparable to similar studies in the literature.^17^^,^^35^^,^^32^ Since the aim of this study was to prove a fundamental effect, we consider this small number of subjects to be acceptable and the influence of the statistical type II or beta error to be tolerable. It should be noted that the six volunteers took part in the study over a total period of 14 weeks and wore the splints with the enamel test specimens a total of 7 times for 2 days each during this period. Overall, the experimental procedure was designed to minimize the variability between the subjects, a disadvantage that can occur in randomized controlled trials.^36^ The use of bovine teeth offers several advantages due to their structural and chemical similarity to human teeth. Human teeth are often extracted for pathological reasons. In addition, study participants may be reluctant to wear parts of foreign human teeth in their oral cavity. These reservations are significantly lower with teeth from cattle that are used for human nutrition and have been tested in veterinary medicine.^37^^,^^38^ Fluorescence microscopy is able to visualize viable and non-viable bacteria, while scanning electron microscopy provides morphological insides into biofilm structures. It is possible to investigate biofilms and the effects of antimicrobial substances.^39^ The LIVE/DEAD® BacLight™ Bacterial Viability Kit (Invitrogen, Molecular Probes, Eugene, Oregon, USA) was employed to stain the test specimens with propidium iodide (PI) and SYTO 9 dyes. While both dyes stain nucleic acids, they differ in spectral properties and membrane permeability: PI is a red fluorescent dye that penetrates damaged membranes, while SYTO 9 can penetrate both intact and damaged membranes, emitting green fluorescence. If both dyes are present, PI displaces SYTO 9 due to its higher affinity for nucleic acids. Fluorescence microscopy has proven reliable, easy to perform, and offers good colour contrast between stained bacteria.^40^ However, there is a correlation between staining capacity and the physiological state of bacteria, with actively metabolizing cells fluorescing more strongly than inactive ones.^41^ Studies suggest that staining may compromise cell viability, a hypothesis confirmed by transmission electron microscopy (TEM), which showed intact bacteria in unstained controls and lysis in stained samples.^41^^,^^42^ Consequently, a processing time of no more than 10 minutes was aimed for when imaging individual test specimens.

Scanning electron microscopy (SEM) was used to confirm fluorescence microscopy results, providing precise structural analysis of biofilms, although it cannot differentiate between viable and non-viable bacteria.^43^ SEM allows magnification up to 20.000 times and was complemented by energy-dispersive X-ray analyses to identify residues from the rinsing solutions.^44^ However, due to the time-consuming specimen preparation and its limitations in assessing bacterial viability, SEM should not be used as the sole evaluation method; instead, it is combined with fluorescence microscopy to enhance biofilm visualization.^45^^,^^43^

The negative control using water exhibited the highest biofilm coverage (71.84 %) and vitality (54.21 %) among all rinsing solutions, indicating the establishment of a robust biofilm, which was confirmed by fluorescence and scanning electron microscopy.^46^^,^^35^ The base paste was also tested as a negative control to evaluate the efficacy of its basic ingredients, revealing no significant differences in vitality (*P* = .69) and coverage (*P* = .13) compared to water, although it tended to have lower biofilm coverage. Scanning electron microscopy also revealed multilayered biofilms during rinsing with the base paste, and the morphology of the bacteria appeared predominantly intact. Compared to the scanning electron microscopic evaluation of the water rinse, a lower coverage was observed, confirming the fluorescence microscopic results. The results thus confirm the efficacy of the base components. These results validate the effectiveness of the base paste, likely due to sorbitol's presence, known for its humectant properties^8^ and inhibitory effects on S. mutans biofilm formation.^47^ However, further research on sorbitol is needed. Overall, the base paste demonstrated greater coverage and vitality than the four tested toothpastes, suggesting the impact of additional ingredients in each formulation.

The 0.2% chlorhexidine (CHX) rinse solution was used as the positive control due to its well-established antibacterial and anti-adhesive properties. It is recognized as the gold standard for controlling biofilms. Rinsing with CHX resulted in the lowest biofilm coverage (9.55%), which is attributed to its anti-adhesive effect. This allows it to bind to negatively charged surfaces and salivary proteins, thereby disrupting pellicle formation.^9^ Although the vitality of bacteria in CHX-treated samples was measured at 38.65%, this value is misleading as the poor coverage resulted in mainly isolated viable bacteria being detected, which complicates the interpretation of the viable-to-nonviable ratio. Scanning electron microscopy confirmed the altered biofilm structure and reduced bacterial integrity, further validating the anti-adhesive properties of CHX observed through fluorescence and electron microscopy. This study confirmed the anti-adhesive effect of CHX using both fluorescence and scanning electron microscopy.

All of the tested toothpastes were effective in reducing both the coverage and vitality of biofilms, achieving reductions of ≤40%. The toothpastes contained various active ingredients, including aluminium lactate (0.8%), chlorhexidine (0.05%), sodium fluoride and sorbitol. Previous studies have confirmed the biofilm-reducing effects of chlorhexidine and aluminium lactate.^33^^,^^9^^,^^10^ This study examined the differences between these ingredient combinations in rinsing solutions, considering dilution effects in situ and without mechanical cleaning. While all toothpastes had a positive effect on reducing biofilms, as evidenced by scanning electron microscopy, there were some differences between them.

The sensitive paste performed slightly better in terms of coverage and vitality reduction than the standard paste, though the difference was minimal for both. The literature on strontium acetate and potassium chloride mainly concerns controlling hypersensitivity rather than affecting the biofilm.^25^^,^^48^ The inclusion of the two additional ingredients in the paste may have contributed to the variation in results. The enzyme paste, which contains papain (0.012%), bromelain (0.05%) and hyaluronic acid (0.2%), achieved the highest vitality (39.21%) of all the pastes and did not demonstrate additional antibacterial effects. The enzyme paste's mean coverage value was comparable to the mean values of the active and sensitive pastes. Bromelain has been shown to interact with the tooth surface in an in vitro study. It reduces the surface tension of bacteria by hydrolysing saliva proteins and glycoproteins, which are bacterial messengers that adhere to the tooth surface.^49^ Another in vitro study demonstrated a growth-restricting effect on Streptococcus mutans, but only at concentrations of 35% or higher.^50^ According to a clinical study by Abdulkareem et al, the mean plaque index can be significantly reduced after using a hyaluronic acid containing mouthwash compared to baseline data.^51^ Another study by Alharbi et al, demonstrated that high-molecular-weight hyaluronic acid can suppress both the formation and growth of P. gingivalis biofilms.^31^ Therefore, hyaluronic acid has a positive effect on oral health and inflammation of the gums and periodontium. It can be concluded that the enzyme paste containing papain, bromelain and hyaluronic acid did not exhibit any additional antibacterial properties in this study. It is unclear whether this is due to insufficient concentration in the toothpastes, possible interaction with, or inhibition by, other ingredients.

The hydroxyapatite toothpaste differed significantly from all the others. Although it allowed the highest biofilm coverage (an average of 40.4%), a strong antibacterial effect was observed. With 9.3%, this solution reduced vitality more than all the other substances tested. This effect is most likely due to the addition of hydroxyapatite. The use of 5% HAP confirms the results of a comparable study by Nobre and Mirela.^52^ Earlier studies only examined and compared HAP and CHX individually.^52^^,^^32^ One possible explanation for the effect of HAP and CHX in combination, as in the present study, is a synergistic relationship between the two substances.

All toothpastes in this study contained 0.05% CHX, with a lower concentration achieved by diluting the suspension and accounting for dilution by saliva. Previous studies have shown that the long-term use of a 0.05% CHX paste containing aluminium lactate has a positive effect on the gingival index with no side effects.^53^ In vitro studies have also demonstrated the antibacterial properties of CHX at concentrations of 0.05% and 0.025%.^54^

Another limitation is the small number of participants. It should be noted that the volunteers had to adhere to a strict oral hygiene regimen, behave in a disciplined manner, and wear splints with test specimens repeatedly over a period of around three months. The number of six volunteers was chosen for biometric reasons, as this is the minimum required for statistical tests. This number was also chosen for ethical reasons, to minimize stress for the healthy test persons, and due to the exploratory nature of this study.

Further studies can then be conducted to specifically investigate the effect of individual active substances or combinations of active substances. Furthermore, further studies should examine the individual components of toothpaste suspensions under mechanical application. In the present study, no sequencing was performed, which further limits the validity of the results. No statement can be made about the quality of the change in the biofilm.

## Conclusions

This in situ study demonstrated the biofilm-inhibiting effect of all toothpastes. The HAP paste containing 5% hydroxyapatite exhibited the most significant antibacterial activity.

## Explanation on the subject of people

The privacy rights of human subjects have been observed and informed consent was obtained for experimentation with human subjects. The studies involving human participants were reviewed and approved by Ethics Committee of the Saarland Medical Association (No. 101/22, amendment: 198/22). All experiments were performed in accordance with relevant guidelines and regulations. Research has been performed in accordance with the Declaration of Helsinki.

## Author contributions

*Stefan Rupf:* Conceptualization, Validation, Writing – original draft preparation, Project administration, Writing – review & editing, Funding acquisition. *Norbert Pütz:* Methodology. *Johanna Dudek:* Methodology. *Lea Lehnertz:* Conceptualization, Validation, Formal analysis, Investigation, Writing – original draft preparation, Visualization, Writing – review & editing. *Madline Priska Gund:* Writing – original draft preparation, Writing – review & editing, Supervision. *Matthias Hannig:* Funding acquisition, Writing – review & editing. All authors have read and agreed to the published version of the manuscript.

## Data availability statement

The data that supports the findings of this study are available from the corresponding author upon reasonable request.

## Conflict of interest

The authors declare the following financial interests/personal relationships which may be considered as potential competing interests: Stefan Rupf, Matthias Hannig reports equipment, drugs, or supplies was provided by Dr Theiss Naturwaren GmBH. If there are other authors, they declare that they have no known competing financial interests or personal relationships that could have appeared to influence the work reported in this paper.

## Appendix

Figure A1
